# Protocol for a randomised controlled feasibility study of psychologically informed vestibular rehabilitation for people with persistent dizziness: INVEST trial

**DOI:** 10.1186/s40814-021-00896-y

**Published:** 2021-08-16

**Authors:** David Herdman, Sam Norton, Marousa Pavlou, Louisa Murdin, Rona Moss-Morris

**Affiliations:** 1 Health Psychology Section, Institute of Psychiatry Psychology and Neuroscience, King’s College London, London, UK; 2 St George’s University Hospitals NHS Foundation Trust, London, UK; 3 Centre of Human and Aerospace Physiological Sciences, King’s College London, London, UK; 4 Guy’s and St. Thomas’ NHS Foundation Trust, London, UK; 5 Ear Institute, University College London, London, UK

**Keywords:** Protocol, Feasibility, Dizziness, Vertigo, Vestibular, Rehabilitation, CBT, PPPD

## Abstract

**Background:**

Dizziness is a common complaint that often persists and leads to disability and distress. Several cognitive and behavioural responses may contribute to the neurobiological adaptations that maintain persistent vestibular symptoms. This paper will present the protocol of a two-arm parallel group feasibility randomised controlled trial designed to determine whether a fully powered efficacy trial is achievable by examining the feasibility of recruitment, acceptability and potential benefits of an integrated cognitive behavioural therapy and vestibular rehabilitation (CBT-VR) treatment for people with persistent dizziness.

**Methods:**

Forty adult patients will be recruited from a tertiary vestibular clinic with persistent movement–triggered dizziness for 3 months or longer who have moderate–high levels of dizziness handicap. Participants will be 1:1 randomised, using a minimisation procedure, to six sessions of either CBT-VR (intervention arm) or VR only (control arm). Measures will be collected at baseline and 4 months post randomisation. The primary feasibility outcomes include descriptive data on numbers meeting eligibility criteria, rates of recruitment, numbers retained post randomisation, treatment adherence and an acceptability questionnaire. Treatment effects on self-report outcomes will be estimated to determine that 95% confidence intervals for the effects are consistent with anticipated effects and minimum clinically important differences, and to provide information needed for the power calculation of an efficacy trial. A nested qualitative study will be conducted post-intervention (intervention group only) to explore the acceptability of the intervention and identify any areas in need of improvement.

**Discussion:**

If a trial of CBT-VR is feasible, acceptability data will be used to enhance the intervention if needed and refine the multicentre RCT protocol. Future studies will need to consider the training required for other physiotherapists to deliver the intervention.

**Trial registration:**

ClinicalTrials.gov, ISRCTN 10420559

**Supplementary Information:**

The online version contains supplementary material available at 10.1186/s40814-021-00896-y.

## Background

Vertigo and dizziness are common complaints in the general population and are often caused by vestibular disorders [1]. Dizziness as a symptom can persist in patients with vestibular disorders even after the recovery of the acute crisis and lead to functional vestibular syndromes [2, 3]. It is frequently accompanied by unsteadiness and a range of other unpleasant and disabling symptoms such as blurred vision, nausea, pallor, psychological complaints, and cognitive deficits in spatial navigation, memory, attention, executive function and body schema [4].

Vestibular rehabilitation (VR) is an exercise-based treatment recommended for people with persistent dizziness and balance symptoms [5]. VR aims to facilitate the ability of the central nervous system to ‘compensate’ and restore normal function [6]. The exercises are based on principles of habituation and adaptation/substitution, in addition to balance retraining [7]. Patients are expected to carry out a home-based exercise programme over a number of weeks or months with graded exposure to dizziness-provoking stimulus as core to the intervention. However, in some of the randomised trials, only around 50% of subjects in the intervention group achieve the desired level of subjective improvement in dizziness symptoms [8]. In clinical practice, around 25% do not improve at all depending on which outcome measure is used, and the majority continue to report ‘bothersome’ symptoms [9].

Since psychological factors are intrinsically linked with recovery from balance disorders, a combination of cognitive behavioural therapy (CBT) and VR has been recommended for a long time now [10]. Indeed, physiotherapists working in vestibular rehabilitation consider managing aspects of anxiety within their scope of practice, but acknowledge the need for tailored training and guidance [11]. Tailored training requires an evidence-based manualised CBT treatment capable of synergistically targeting mental and physical health aspects of dizziness. In a systematic review, four randomised controlled trials (RCTs) reported improvement in dizziness following therapy, combined with VR or relaxation techniques [12]. However, the sample sizes were small, and the effects on dizziness outcomes tended to be weak, with one study evaluating long-term effects finding similar results to those obtained before treatment [13]. The components of the therapy were not described in detail and did not involve a strict manual.

Since then, Edelman et al. [14] found reductions in dizziness outcomes, avoidance and safety behaviours, but not depression or anxiety in a short 3-session psychological intervention compared with a waiting list control. These effects were maintained after 6 months, although higher levels of anxiety predicted higher levels of disability [15]. A recent feasibility study evaluated a group intervention based on traditional VR and a model of CBT based on panic anxiety. Only one participant experienced a meaningful improvement in pre and posttreatment scores on the subjective dizziness outcome suggesting CBT based on panic, and/or group-based treatment may not be the best protocol [16].

These studies highlight that there is no agreed theoretical framework or manualised treatment protocol, which sufficiently integrates the psychological and self-management needs of patients with chronic dizziness. This makes it difficult to replicate interventions but also raises important theoretical implications when CBT protocols are based on empirical cognitive–behavioural models of depression and anxiety. In these models, emotions are conceptualised as primary mental health disorders rather than a reaction to objectively challenging symptoms. These protocols also fail to address illness-specific behavioural self-management techniques.

For individuals experiencing persistent dizziness, a CBT protocol which remains contextually anchored to their experience of living with dizziness may ultimately promote better engagement with rehabilitation and improve health outcomes. We conducted a theoretical modelling prospective study which revealed the importance of a variety of illness-specific cognitive and behavioural factors in the experience of dizziness-related disability [17, 18]. This was drawn together with a review of the literature to develop a model specific to dizziness (article in preparation), and we then used intervention mapping techniques [19, 20] to design an intervention and detailed manual which integrated CBT methods into traditional VR.

The aim of this study is therefore to evaluate the feasibility of the manualised ‘INVEST’ (integrated CBT-VR) protocol, for participants with persistent dizziness, as part of the preparation for a full-scale randomised controlled trial.

### Primary objectives

The following are the primary objectives:

1. To determine the recruitment rate
2. To assess retention of participants by estimating follow-up rates
3. To assess the acceptability of the intended self-report outcome measures for a future definitive trial (i.e. questionnaire feedback, completion rates, item-level missing data, floor/ceiling effects and estimates of variance)
4. To explore the level of acceptability of the interventions through a survey and by measuring percentage of patients completing each of the interventions
5. To formulate a suitable method to measure physiotherapist fidelity for a future multicentre trial

### Secondary objectives

The following are the secondary objectives:

1. To explore treatment effects on self-report outcomes to determine that 95% confidence intervals for the effects are consistent with anticipated effects and minimum clinically important differences
2. To estimate key elements that would inform a power calculation to inform a power calculation for an efficacy study
3. To qualitatively explore patient perceptions of the credibility, acceptability and usefulness of the intervention and identify areas of improvement for a future full-scale trial

## Methods

### Design

This feasibility randomised control trial with nested qualitative study will be composed of two-armed, parallel groups, to gather preliminary information on the intervention (INVEST) and the feasibility of conducting a full-scale trial.

### Setting

Participants will be recruited and treated at the audio-vestibular and physiotherapy service at St George’s University Hospitals NHS Foundation Trust. Questionnaires and outcome assessment will be done online.

### Sample size

In agreement with current recommendations for pilot study sample size, 20 participants will be included in each group [21, 22]. Given a sample size of 40, assuming participation rates of 33% and drop-out rates of 20%, it will be possible to estimate 95% confidence intervals for the participation and drop-out rates within a maximum interval of ± 9% and ± 16% respectively.

### Participants

Participants will be recruited who must meet all the following criteria:

- Patients attending the neuro-otology balance clinic at St George’s University Hospitals Foundation Trust with symptoms of chronic dizziness (≥ 3 months) made worse by movement of the self and/or the environment
- Have a vestibular diagnosis^1^
- Dizziness Handicap Inventory (DHI) ≥ 40
- Aged ≥ 18 years
- Not currently participating in vestibular rehabilitation or psychological treatment (talking therapies)
- Able to provide consent and willing to comply with the proposed training and testing regime

Participants will be excluded if they meet one or more of the following exclusion criteria:

- Patients with vestibular migraine or other headache/migraine disorder with ≥ 3 headaches a month and/or MIDAS (Migraine Disability Assessment) ≥ 6 since they would not usually be suitable for vestibular rehabilitation until their headaches are under control
- Patients with active Meniere’s disease or BPPV (benign paroxysmal positional vertigo) since they would not usually be suitable for vestibular rehabilitation until their vestibular function is stable
- Patients with central vestibular disorders (excluding migraine and functional disorders), other neurological disorders, bilateral vestibulopathy or acute severe mental health illness since these conditions would interfere in the outcome of rehabilitation
- Patients with acute orthopaedic disorders influencing balance control and gait
- Insufficient grasp of written/spoken English or have special communication needs

### Flow of recruitment and participant timeline

**Fig. 1:**
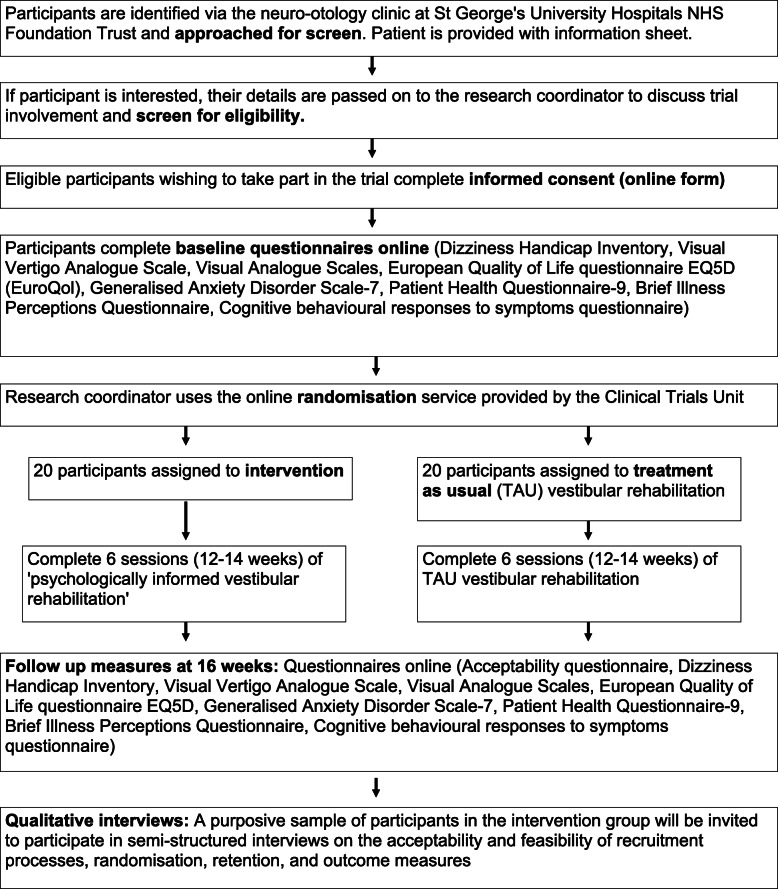
Trial flowchart

Patients will be approached to participate by the Audiovestibular Physicians in the vestibular clinic who will complete the initial screen (see Fig. 1). Interested potential participants will be given a participant information sheet and contacted by the principal investigator (DH) for telephone screening to make sure they meet all the inclusion criteria (e.g. DHI criteria). Consent forms and baseline questionnaires will be completed online. Participants will then be randomised to either the intervention group or the control group. Follow-up data will be collected at 4 months post randomisation, and data will be anonymised. On completion of the postintervention measures, a subsample of participants will be invited to take part in the qualitative interview.

### Randomisation and blinding

Participants will be randomised consecutively, and physicians will be blinded to allocation sequence. Randomisation will be completed by the King’s Clinical Trials Unit via an online electronic system using a minimisation procedure with a probability of 0.8 to assure similar distribution of selected participant factors between trial groups, to include three dichotomous outcomes: gender (male/female), age (18–60/over 60) and dizziness handicap (DHI score 40–59/≥ 60).

### Interventions

#### INVEST intervention

The treatment is a tailored integrated cognitive behavioural therapy–vestibular rehabilitation (CBT-VR)–based intervention with therapist support. The purpose of this intervention is to target individual’s dizziness beliefs and cognitive–behavioural responses to symptoms in order to facilitate vestibular rehabilitation. The development of the intervention was systematic, based on findings of a review and prospective studies, with substantial input from patient and public representatives and a multidisciplinary team of health psychologists, physiotherapists and audiovestibular physicians. The structure and content of the manual was drafted based on previous CBT interventions developed by the department of Health Psychology at King’s College London [23–25], and other sources [26–28].

Participants will be provided with a structured therapy manual including worksheets. This will be accompanied by six sessions with the primary researcher (DH) who has experience in working with patients with severe dizziness as a physiotherapist and has received some basic training in CBT. In accordance with CBT-VR principles, participants will be encouraged to complete tasks and exercises between sessions. The first session will be structured around education and include an individual formulation and cognitive behavioural analysis of the dizziness problem. The general point of the first session is that the patient’s behavioural responses are a normal defensive response to the aversive stimuli, which may have been adaptive in acute dizziness but have lost their efficacy as the dizziness has persisted.

The participant is guided towards sections of the manual that may be more relevant to their own problem. It includes the following components:

1. Education: Educational content about persistent dizziness is provided, and participants are given space to develop their own case formulation to help make sense of their experiences from a psychophysiological perspective.
2. Goal setting: Worksheets allow participants to set goals for therapy. Specific functional goals are encouraged that redirect the focus of attention toward daily life activities and are broken down into achievable steps.
3. Activity monitoring: Worksheets help participants to identify avoidance and/or all-or-nothing behaviours, establish activity tolerance levels and identify discriminative stimuli eliciting dizziness behaviours. Participants are encouraged to adopt a consistent and balanced approach to activities through planning activity diaries.
4. Distraction techniques: Education about distraction with in-session behavioural experiments to demonstrate the effects of symptom focusing and attention switching on dizziness and balance
5. Reattribution of symptoms: Participants are encouraged to identify symptoms and reattribute them to either symptoms of their condition, medication, deconditioning, stress and anxiety or depression.
6. Relaxation techniques: The link between autonomic anxiety and dizziness is presented and relaxation methods introduced including diaphragmatic breathing, progressive muscle relaxation and guided imagery relaxation.
7. In vivo exposure: Participants identify avoidance and safety behaviours and establish a dizziness-related fear hierarchy followed by graded exposure to fear eliciting activities in a series of behavioural tests during which catastrophic expectations are challenged.
8. Cognitive therapy: The link between thoughts and behaviours is presented, participants encouraged to identify their own thoughts, and worksheets to restructure the dizziness-related beliefs and behavioural experiments designed to challenge maladaptive beliefs.
9. Problem solving: A review of information and strategies implemented so far and review of progress are made with additional information on fear beliefs, perfectionism, managing financial and work related stress, and sleep problems. Sleep restriction therapy is recommended where appropriate.
10. The potential for dizziness flare-ups is managed proactively by attempting to alter the patient’s expectations and reduce the likelihood of catastrophising throughout therapy. The patient reflects on progress made over the 6 sessions and develops a relapse management toolkit.

Although originally, all sessions were designed to be face to face, to be consistent with current service provision due to the COVID-19 pandemic, we will not discriminate against people who cannot attend in person, and instead offer them the same therapy over video consultation software.

The first session will last 1 h, while the remaining five sessions will last 30 min. This is consistent with current physiotherapy clinical practice. Table 1 includes a summary of content for the sessions. As a general rule, participants may need sessions once a fortnight initially, but the time between sessions becomes more spaced out as therapy progresses, and they become more independent, for 12–14 weeks.

**Table 1:** Summary of content for the sessions

Summary of sessions	
Session	Content
1	Understanding the problem (formulation) Familiarisation with workbook Symptom control techniques Homework: activity monitoring
2	Review activity diary Goal setting Physiotherapy exercises Activity planning Homework: activity and rest goal setting
3	Review sleep, activity and rest goal sheet In vivo behavioural experiments Homework: behavioural experiments & exposure training
4	Review homework Review of beliefs and cognitions Progress physiotherapy exercises Homework: thought diary
5	Review thought diary Review of progress and problem solving Homework: depending on identification of ongoing problems (e.g. sleep therapy, etc.)
6	Planning for the future Relapse management

#### Treatment as usual

Treatment as usual will be vestibular rehabilitation, consisting of specific exercise techniques to target identified impairments or functional limitations, delivered by a senior specialist vestibular physiotherapist at St George’s Hospital. The physiotherapy will be consistent with the latest evidence-based Clinical Practice Guidelines [29]. Participants will also be asked to complete a home exercise programme. The session duration and schedule will be the same as the intervention with the first session lasting an hour and follow-up appointments 30 min, up to six sessions between 12 and 14 weeks.

### Clinical supervision

DH has attended training to deliver low-intensity CBT techniques and will undergo further training with role-played sessions with feedback from RMM. Ongoing supervision will be provided by RMM. Shared reflection of recorded sessions will be discussed in line with the core competency framework for delivering psychological therapies in long-term conditions [30].

### Intervention fidelity

The therapist delivering the intervention sessions will follow the detailed and structured manual developed for the patients. With permission from the participants, sought on the consent form, therapy sessions will be video recorded and assessed for fidelity during supervision by RMM.

### Primary feasibility outcomes

Feasibility will be assessed by collecting descriptive data on recruitment and retention rates and willingness to be randomised according to the Consolidated Standards of Reporting Trials feasibility and pilot trial guidelines [31]. The following will be recorded:

- Suitability of eligibility criteria: number of people excluded from the trial and for what reasons. This will allow us to assess whether the criteria are appropriate.
- Willingness to participate: the proportion of eligible patients who agree to participate
- Retention rates: the proportion of participants who were randomised that completed follow-up assessment as well as recording of attendance at therapist sessions. If participants drop out, we will attempt to contact them to find out the reasons.
- Time needed to collect and analyse data: time sheets will record the duration of collection and analysis of the data.
- Acceptability/satisfaction of the intervention: This will be evaluated at follow-up using a questionnaire based on the component constructs in the theoretical framework of acceptability [32]. It will take the form of eight statements using a five-item Likert response scale (strongly agree to strongly disagree):
  - I feel positive about the treatment.
  - The amount of effort required to participate in the treatment was acceptable.
  - The treatment fits with my values.
  - The treatment made sense to me.
  - The time involved in engaging in the treatment was acceptable to me.
  - The treatment was effective to help me manage my condition.
  - I was able to perform the activities required to participate in the treatment.

### Self-report outcomes

#### Dizziness handicap

The Dizziness Handicap Inventory (DHI) [33] consists of 25 questions designed to assess physical, functional and emotional aspects of dizziness-associated disability and ‘handicap’. For each question, the participant can choose ‘yes’, ‘no’ or ‘sometimes’, and the total score ranges from 0 to 100 with higher scores indicating more severe handicap and activity restriction. With high test–retest reliability and low error of measurement scores, the DHI has been widely adopted in clinical practice and trials to evaluate the effects of vestibular rehabilitation with mixed dizziness diagnoses [33–35].

#### Visually induced dizziness

The Visual Vertigo Analogue Scale (VVAS) [36] is a nine-item visual analogue scale that rates the intensity of dizziness during daily situations typically inducing ‘visually induced dizziness’ (ViD) such as ‘walking through a supermarket aisle’ or ‘watching action television’. Intolerance of visual motion is a common symptom for people with chronic vestibulopathy induced by dynamic visual input and has been shown to be a negative prognostic indicator [2, 37]. The VVAS shows validity and responsiveness to change [38].

#### Dizziness interference

Dizziness interference will be calculated using a visual analogue scale. Participants will answer the question, ‘Over the past week, what percentage of the time has dizziness interfered with your activities?’ by drawing a vertical line across a 10-cm line with 20% increments. Test–retest reliability for this tool is excellent [39].

#### Health-related quality of life

The European Quality of Life questionnaire EQ5D (EuroQol) [40] measures health-related quality of life for clinical and economic appraisal. The first part of the instrument is a self-reported description of the subject’s health using a five-dimensional classification. It contains five items, each with three response choices. The answers are converted into a score ranging up to 1.00, indicating high health-related quality of life. The second part is a self-rated valuation of the subject’s health using a vertical VAS in the form of a thermometer ranging from 0 (worst imaginable state of health) at the bottom to 100 (best imaginable state of health) at the top. The test–retest and inter-rater reliability has been established for patients with dizziness and disequilibrium [41] and has been used to assess cost effectiveness of vestibular rehabilitation [42].

#### Balance

The trial register entry (ISRCTN 10420559) includes a blinded mini-Balance Evaluation Systems Test (mini-BESTest) [43, 44]. Due to the COVID-19 local restrictions, it is no longer possible for participants to attend in person for this test, and it has therefore been removed from the protocol. When possible, the physiotherapists will be encouraged to complete and record this assessment as part of their initial evaluation.

### Self-report outcomes: process variables

The following self-report outcomes will be targets for the intervention so will also be assessed:

#### Illness perceptions

The Brief Illness Perception Questionnaire (B-IPQ) [45] is a nine-item scale where each item assesses one dimension of illness perceptions. In accordance with the recommendations from the authors, the word ‘illness’ will be replaced by ‘dizziness condition’ in order to reflect the specific dizziness illness–related perceptions of participants. It may be possible to compute an overall score which represents the degree to which the illness is perceived as threatening or benign. The internal consistency of this score will be checked.

#### Cognitive and behavioural responses to dizziness

The Cognitive and Behavioural Responses to Symptoms Questionnaire (CBRQ) [46] assesses patients’ cognitive and behavioural responses to the experience of symptoms. The five subscales dealing with cognitive responses are symptom focusing (e.g. ‘I think a great deal about my dizziness’), catastrophising (e.g. ‘I will never feel right again’), damaging beliefs (e.g. ‘dizziness is a signal that I am damaging myself’), fear avoidance (e.g. ‘I should avoid exercise when I have dizziness’) and embarrassment avoidance (e.g. ‘The embarrassing nature of my dizziness prevents me from doing things’). The two behavioural subscales are all or nothing (e.g. ‘I find myself rushing to get things done before I crash’) and avoidance/rest (e.g. ‘I stay in bed to control my dizziness’). High scores indicate more unhelpful responses, and the reliability and validity have recently been established for patients with dizziness [47].

#### Anxiety and depression

Depressive symptoms will be assessed using the Patient Health Questionnaire-9 (PHQ-9) [48], and anxiety will be assessed using the Generalised Anxiety Disorders-7 Questionnaire (GAD-7) [49]. These questionnaires have been widely validated in physically ill populations, and higher scores indicate more severe symptoms.

### Other treatments

Participants will be asked whether they have received any pharmacological, psychological or exercise-based treatment in addition to INVEST since starting the study.

### Adverse events

Information about occurrence of serious adverse events since the start of the study will be reported according to good clinical practice guidelines. Adverse events will be flagged up to the trial management team, and participants will be contacted to further assess the adverse event and its relationship to the study.

### Sociodemographic and clinical characteristics

Sociodemographic characteristics including gender, age, ethnicity and level of education will be collected at baseline via self-report. Clinical characteristics will include the diagnosis and will be verified at baseline according to their clinical records. The clinical diagnoses will be made by an Audiovestibular Physician based on the Barany diagnostic criteria.

### Qualitative interviews

Qualitative methods will be used in order to obtain a more comprehensive understanding of acceptability of the trial requirements and therapy approach, therapy outcomes and feedback on the intervention.

The sample will be recruited from the feasibility trial, and the study will be nested within the main trial. Participants will be asked for additional consent to be interviewed. When each of these participants completes their trial intervention and their post-therapy assessment, a decision will be made as to whether to contact them for interview. After the first 10 interviews, sampling will become increasingly purposive with the aim of interviewing a sample with maximum variation. We will seek variation in terms of demographics, and attitudes towards therapy as gleaned from responses to Likert scale questions described previously. The sample will not be selected to be representative of the trial participants but to include people likely to hold different viewpoints.

Interviews will be scheduled as soon as possible after completion of the post-therapy questionnaire assessments. The interview will consist of a series of open-ended questions relating to expectations of the interventions, how participants found the therapy and any changes they had experienced. All interviews will be recorded and transcribed verbatim.

### Analysis plan

Descriptive statistics of patients approached, screened, eligible, consented and randomised will be computed to address the primary objectives. Reasons for non-consent, exclusion and drop-out, at each stage of the study, will be recorded and reported. Adherence to the intervention will be reported using descriptive statistics to include the mean number of sessions completed, a breakdown on the number of participants completing each session and mean duration of the sessions. To account for uncertainty due to sampling error, all estimates will be presented with 95% CIs. The standard deviation of the key self-reported outcome and the correlation between the baseline and follow-up assessments of the outcomes will be computed to inform the sample size for a future efficacy trial.

To address acceptability, a mixed methods approach will be used, drawing on both the quantitative and qualitative findings to determine any intervention-specific issues, including whether the number and pacing of sessions seemed sufficient.

The psychometric adequacy of the self-report instruments used will be assessed to address the secondary outcomes. Floor and ceiling effects will be considered as a key indicator of potential sensitivity of the scale to detect changes. Reliability will be assessed using Cronbach’s alpha, with a minimum acceptable cut-off at *α* = 0.70, but preferably at *α* = 0.80 or higher, particularly for the key variables. Non-completion of individual items will be checked to ensure that there are no potentially problematic items for this patient population.

An analysis of covariance (ANCOVA) approach will be performed to estimate the postintervention mean difference in outcomes: dizziness disability (handicap), dizziness severity, dizziness interference, depression and anxiety. Given the feasibility nature of the trial, the statistical significance of any post-randomisation group differences will not be assessed; instead, effect sizes and CI will be estimated and used for interpretation. Each analysis will adjust for the baseline level of the outcome variable and factors used in the minimisation procedure. Group allocation will be included as an indicator variable following the intention-to-treat principle.

Finally, to qualitatively explore the acceptability and usefulness of the intervention from the perspective of the participants, the semi-structured qualitative interviews will be transcribed verbatim and analysed using inductive thematic analysis with the use of NVivo software. Thematic analysis revolves around identifying recurrent themes and patterns from the interviews and developing a coding manual [50].

### Progression criteria

To inform the decision whether to proceed to a full-scale efficacy trial, the following a priori criteria will be used. We will deem the trial appropriate to progress if (1) ≥ 70% of eligible patients participate; (2) drop-out rate is < 20%; (3) there are comparable acceptability ratings to the TAU based on the quantitative and qualitative data and (4) ≥ 60% adhere to sessions. The ‘Stop’ criteria will consider if (1) < 30% of eligible patients participate; (2) drop-out rate is > 40%; (3) < 60% of participants report acceptability of the intervention according to quantitative and qualitative data and (4) < 30% adherence to sessions. Stop criteria will also consider if there are irreconcilable serious adverse events attributed to the intervention (e.g. due to behavioural experiments, in vivo exposure, etc.). Where the assessment outcome falls between the ‘Go’ and ‘Stop’ criteria, the trial committee will consider the data and identify steps needed to progress to a full-scale trial. The trial committee will consider the data presented and make a judgement about whether the methodology and intervention were delivered as intended. We will also use the experience from clinicians and participants to further optimise the intervention and manual.

## Discussion

In recent years, there has been a greater demand to integrate cognitive approaches and enhance the behavioural aspects of vestibular rehabilitation. This protocol represents such an integrated treatment designed specifically to manage the problems associated with the maintenance of persistent dizziness. It represents a theory driven and scientific approach designed following the Medical Research Council guidance [20] for developing and evaluating complex interventions. It has also been designed to be delivered by vestibular physiotherapists, which offers a pragmatic solution to the problem accessing psychological treatment interventions tailored to the specific problems associated with dizziness.

This study is limited because it is single site and includes only one treating therapist in the intervention arm. Future studies will need to consider the training required for other physiotherapists to deliver the intervention. There may be restrictions on participants attending in person due to the current pandemic. As a first step, this study will identify unique challenges that occur in the recruitment and retention of patients and will be able to examine the acceptability of this treatment to patients in terms of whether its content was relevant and useful. This will allow the researchers to further refine the intervention, consider the most suitable training needs for therapists, and substantially inform the design of a future large-scale trial powered to detect the efficacy of integrated CBT and VR treatments for the management of persistent dizziness, accompanied by a longer follow-up to assess any sustained effects of the intervention on outcomes.

## Supplementary Information

**Additional file 1.** SPIRIT 2013 Checklist.

## Data Availability

Not applicable

## Abbreviations

ANCOVA: Analysis of covariance
B-IPQ: Brief Illness Perception Questionnaire
CBRQ: Cognitive and Behavioural Responses to Symptoms Questionnaire
CBT: Cognitive behavioural therapy
DHI: Dizziness Handicap Inventory
EQ5D (EuroQol): The European Quality of Life questionnaire
GAD-7: Generalised anxiety disorders–7
Mini-BESTest: The mini-Balance Evaluation Systems Test
MIDAS: Migraine Disability Assessment
PHQ-9: Patient Health Questionnaire–9
TAU: Treatment as usual
ViD: Visually induced dizziness
VR: Vestibular rehabilitation
CBT-VR: Cognitive behavioural therapy and vestibular rehabilitation
VVAS: The Visual Vertigo Analogue Scale

## Footnotes

1 The early trial register stated that patients with ongoing investigations would be excluded. Due to the current restrictions on vestibular testing during the COVID-19 pandemic, we will not exclude patients based on this criterion. Nevertheless, we still expect the Audiovestibular Physicians to make a reasonable clinical diagnosis based on the Barany classification and to defer recruitment should investigations be essential to make a diagnosis.
